# Modeling the Basal Dynamics of P53 System

**DOI:** 10.1371/journal.pone.0027882

**Published:** 2011-11-16

**Authors:** Tingzhe Sun, Weiwei Yang, Jing Liu, Pingping Shen

**Affiliations:** State Key Laboratory of Pharmaceutical Biotechnology, Department of Life Sciences, Nanjing University, Nanjing, Jiangsu Province, People's Republic of China; German Cancer Research Center, Germany

## Abstract

**Background:**

The tumor suppressor p53 has become one of most investigated genes. Once activated by stress, p53 leads to cellular responses such as cell cycle arrest and apoptosis.

**Methodology/Principal Findings:**

Most previous models have ignored the basal dynamics of p53 under nonstressed conditions. To explore the basal dynamics of p53, we constructed a stochastic delay model by incorporating two negative feedback loops. We found that protein distribution of p53 under nonstressed condition is highly skewed with a fraction of cells showing high p53 levels comparable to those observed under stressed conditions. Under nonstressed conditions, asynchronous and spontaneous p53 pulses are triggered by basal DNA double strand breaks produced during normal cell cycle progression. The first peaking times show a predominant G1 distribution while the second ones are more widely distributed. The spontaneous pulses are triggered by an excitable mechanism. Once initiated, the amplitude and duration of pulses remain unchanged. Furthermore, the spontaneous pulses are filtered by ataxia telangiectasia mutated protein mediated posttranslational modifications and do not result in substantial p21 transcription. If challenged by externally severe DNA damage, cells generate synchronous p53 pulses and induce significantly high levels of p21. The high expression of p21 can also be partially induced by lowering the deacetylation rate.

**Conclusions:**

Our results demonstrated that the dynamics of p53 under nonstressed conditions is initiated by an excitable mechanism and cells become fully responsive only when cells are confronted with severe damage. These findings advance our understanding of the mechanism of p53 pulses and unlock many opportunities to p53-based therapy.

## Introduction

Tremendous efforts have been focused on the homeostatic control of physiological processes. The delicate control over homeostasis plays pivotal roles in maintaining the integrity of cellular structure. When confronted with detrimental signals, cells must initiate a program that preserves the genome or leads to protective apoptosis to eliminate adverse cells. On the other hand, intrinsically transient stress such DNA damage originated from cell cycle progression and physiological reactive oxygen species should not invoke a devastating and frequent cell death in order not to compromise the physiological homeostasis. Therefore, a critical question is raised as how normal cells retain high sensitivities to severe damage while tolerate intrinsically spontaneous damage.

Recent advantage has identified the tumor suppressor p53 as a central node within a vast network that regulates homeostasis [1]–[3]. P53 is a transcription factor that dictates numerous progresses in normal cell progression. The importance of p53 pathways is well demonstrated by the fact that nearly all cancers show defects in this system and nearly 50% harbor mutations in p53 genes [1]. Thus, the dynamic control of p53 levels will tip the balance between survival and death. P53 regulates the expression of multiple proteins and forms many feedback loops. An outstanding one is p53-MDM2 negative feedback loop [4]. P53 can transcriptionally activate MDM2 expression and MDM2 can further targets p53 for proteosome degradation [4]–[5]. P53-MDM2 feedback loop together with another characterized negative feedback loop involving WIP1/ATM/Chk2 forms the basis of p53 pulses and previous experiments have confirmed sustained p53 pulses under stressed conditions [6]. However, not until recently has great progress been made by Loewer *et al* that how cells discriminate physiologically spontaneous and externally severe damage [7]. They quantified the basal dynamics of p53 and found that under nonstressed conditions, spontaneous p53 pulses are triggered through an excitable mechanism. P53 is retained in a transcriptionally latent form and cannot induce p21 under nonstressed conditions. Once challenged by DNA damage inducing agents, further acetylated p53 is competent of inducing p21 and initiates cell cycle arrest [7]. These important findings have shed resplendent lights on how the stress responsive p53 pathway coordinates sensitivity and tolerance during normal cell progression.

However, a theoretical exploration is still lacking as few models evaluated the basal dynamics of p53. Most previous models keep p53 at low steady state (in deterministic equations) and invoke pulses or oscillations on exposure to external DNA damage signal [8]–[16]. The prominent work by Loewer *et al*. challenged previous model scheme by unraveling the basal p53 pulses under nonstressed conditions [7]. Therefore, a new theoretical model should be proposed to further characterize the basal dynamics of p53.

**Figure 1:**
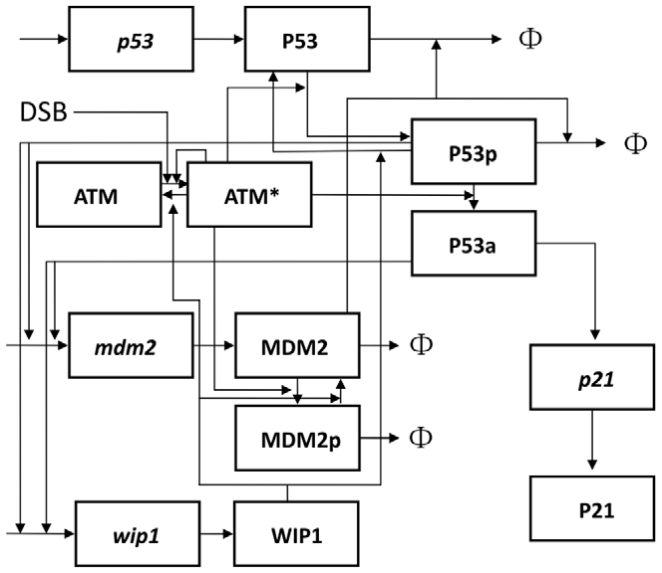
Schematic representation of p53 network. Italics represent mRNA species and Φ denotes degradation.

In current study, we aim to mathematically model the basal dynamics and functions of p53 pulses and unravel the underlying mechanism. We developed a simplified p53 model that covers essential feedback loops in p53 network (The schematic diagram is shown in Figure 1. For detailed description, please refer to materials and methods section). In order to investigate the stochasticity of p53 pulses, we performed a stochastic delay simulation based on binomial τ-leap method. We also incorporated transcriptional bursts in our stochastic simulation. We first investigated whether the model was consistent with previous experiments both qualitatively and quantitatively. In deterministic simulations, we found that either reduced or overexpressed WIP1 can diminish the shape of sustained p53 pulses. We also showed that the pulse period is more concise while the amplitude is highly variable. In stochastic simulations, we further identified that although most cells under nonstressed conditions show low levels of p53 as expected, some cells showed high levels comparable to the levels in stressed condition and therefore, the protein distribution is highly skewed. Furthermore, we elucidated a predominant first pulse distribution at G1 phase of cell cycle which is well consistent with experimental results. Subsequent analyses revealed an excitable mechanism which is qualitatively accordant with experiments. Finally, we found basal activation of p53 pulses is filtered and *p21* levels remain at low state. Either exposure to external stress or inhibited deacetylation can lead to high levels of *p21* transcription.

## Results

### The deterministic system shows sustained pulses

**Figure 2:**
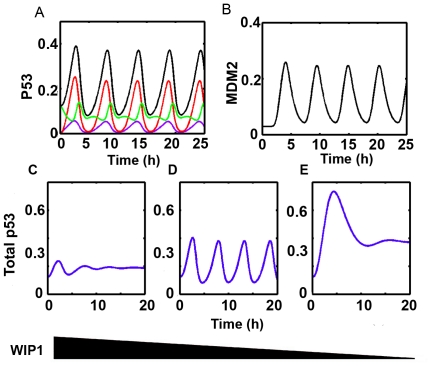
Deterministic dynamics of p53 and MDM2. (A) Total p53 (black), phosphorylated p53 (violet), acetylated p53 (fully competent form, red), unmodified p53 (green). (B) MDM2 pulses. (C–E) p53 dynamics with different WIP1 levels. WIP1 levels decrease from left to right (C: overexpression, s_wip1_∶0.0045 and e_2_∶0.05. D: nominal E: reduced expression, s_wip1_∶0.001 and e_2_∶0.001).

Before performing stochastic simulations, our first attempt is to verify that the dynamic properties of p53 pulses can be reproduced. Nominal parameters were used and DSB was set to be 300 (i.e. approximately 10 Gy γ-irradiation). Figure 2A shows the response of p53 when system is challenged by DNA damage. We found that the system ignites sustained and undamped p53 pulses when exposing to DNA damage (Figure 2A). Total MDM2 also show pulsatile dynamics although there is a differentiable delay (Figure 2B). In bifurcation analysis, we found that the system undergoes a supercritical Hopf bifurcation on crossing the critical points [0.0205 and 0.3019 respectively], Figure S1). The period of the pulses is near 5 hours which is consistent with experimental reports [7], [17].

### WIP1 levels regulate the uniform shape of p53 pulses

Recently, Batchelor et al identified the critical role of WIP1 in maintaining the uniform shape of p53 pulses [6]. Either overexpression or reduction will diminish the undamped pulses [6]. Therefore, we performed in silico experiments to see whether the characteristic pulses could be disrupted when WIP1 is abnormally expressed. First, we *in silico* ‘knocked down’ WIP1 levels by lowering the induction rates (s_wip1_∶0.001 and e_2_∶0.002). We found that the p53 pulse is damped and sustainable shape is lost (Figure 2C) compared with the unperturbed system (Figures 2A and 2D). Then, we elevated WIP1 levels by assigning high induction rates of *wip1* (s_wip1_∶0.0045 and e_2_∶0.05). Similarly, deregulated WIP1 jeopardizes the sustained pulses (Figure 2E). Meanwhile, the mean level of p53 is also increased compared with nominal system (compare Figure 2D and 2E). This is consistent with experiments which show that removing WIP1 leads to increased p53 levels [6].

### Periods are concise and amplitudes are relatively variable

**Figure 3:**
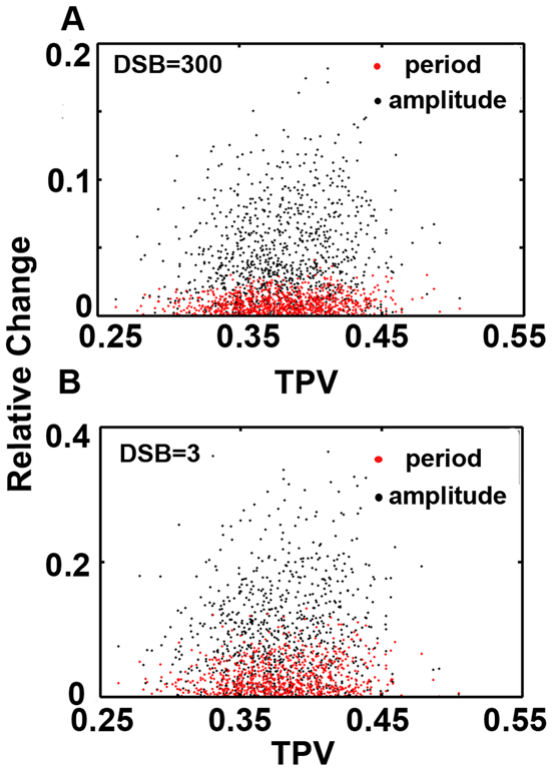
Amplitude and period variations. (A) DSB = 300 (B) nonstressed condition. (amplitude: black, period: red).

On next step, we set out to determine whether some properties of p53 pulses can be qualitatively verified in the model. We first generated 1000 random parameter sets with 5% perturbed from the reference set. Then we integrated the deterministic system. We found substantial fluctuations in amplitude while period is less fluctuated (Figure 3A). To exclude the possibility that this property is damage specific, we further evaluated the variations by taking a lower damage. Similarly, the dynamic property is conserved with respect to DNA damage levels (Figure 3B). Geva-Zatorsky *et al.* found that the amplitude of pulses are more variable than period [17]. Our deterministic model is qualitatively consistent with experiments. Noticeably, unlike the case with higher DNA damage (i.e. 0%), some perturbed parameter sets do not lead to sustained pulses when the damage level is relatively low (∼14%, Figure 3B, i.e. there are only ∼86% points compared to Figure 3A).

Taken together, simulations suggested that at least some properties of p53 pulses were captured in our deterministic model. To some extent, the (at least partial) consistency prompted us to further stochastic simulations.

### Identifying the p53 pulses under nonstressed condition

**Figure 4:**
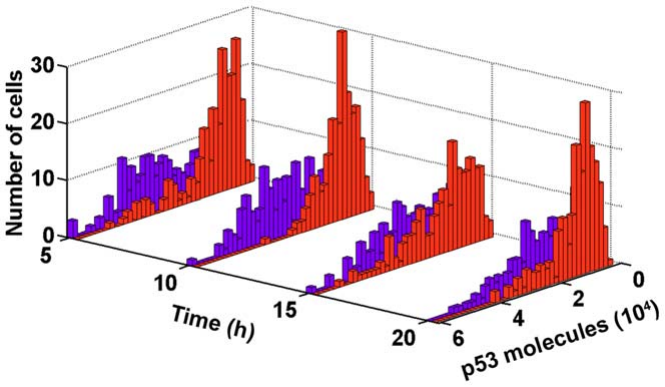
P53 protein distribution. Stressed condition (DSB = 300, violet), nonstressed condition (red).

To identify the basal dynamics of p53, we first quantified the p53 dynamics under nonstressed condition (see materials and methods). We performed stochastic simulations (200 samples). The sampling number 200 was chosen because in experiments, the recorded number of single cell fluorescence is from tens to over 100 [7], [17]. Then we collected the p53 levels at four different times to simulate the experimental settings (i.e. immunofluorescence experiments in [7]). Astonishingly, p53 levels were not uniform across all simulations (Figure 4, red bars). Most cells showed low levels of p53, but in most situations, there was a significant fraction of cells that showed high p53 levels comparable to those observed under stressed conditions (Figure 4, compare red and violet bars, stressed condition: DSB = 300. Endogenous DSB production, i.e. stochastic formulation of spontaneous DSB production as described in materials and methods section, is halted because cells undergo G1 arrest). In other words, the protein distribution was highly skewed (i.e. right tailed).

**Figure 5:**
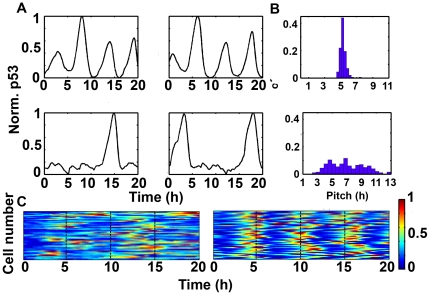
Stochastic p53 dynamics under stressed and nonstressed conditions. (A) Dynamics of p53 under stressed (top panels) and nonstressed condition (bottom panels). (B) Characteristic pitch distributions. Stressed (top), nonstressed (bottom). (C) 50 samples of p53 dynamics (normalized, left: nonstressed, right: stressed).

There are two possibilities that can explain the observed variations. First, p53 is kept at low levels under most circumstances and small fraction of cells has high steady levels. A second probability argues that the levels of p53 are dynamically changed. To discriminate these two scenarios, we quantified individual simulation. When challenged with DNA damage, p53 showed undamped pulses (Figure 5A, top panel). Surprisingly, under basal nonstressed conditions, most cells exhibited at least one p53 pulse (Figure 5A, bottom panel). The characteristic period (i.e. pitch, the time of the first maximum of the autocorrelation function) of cells implied that p53 pulses under nonstressed condition were irregular and asynchronous (Figure 5B, compare top and bottom panel). The distribution of interspike intervals also shows that the basal pulses are highly variable (Figure S2). We randomly collected 50 trajectories of p53 pulses from both stressed and nonstressed conditions and found that synchronicity was lost under basal unstressed condition (Figure 5C).

These results showed that in response to DNA damage, the dynamics of p53 shifts from series of spontaneous, asynchronous pulses to regular and synchronous pulses. Our simulation showed accordance with experimental results [7].

### Basal p53 pulses exhibit cell cycle dependence

**Figure 6:**
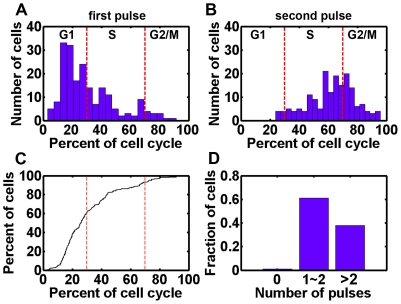
Pulse correlation with cell cycle. (A and B) Distribution of first (A) and second peaking times. Dashed lines are guide lines for different cell cycle phase. (C) Cumulative distribution of first peaking times. Dashed lines are guide lines for cell cycle phase. (D) Number of pulses in one cell cycle.

We have developed a simplified DSB repair module which incorporated spontaneous DSB production during normal cell cycle progression (see materials and methods for the definition of different cell cycle phases). We synchronized cell cycle *in silico* as described in materials and methods. We found that the p53 pulses are correlated with cell cycle phase with a predominant G1 distribution for first peaking time (60.05%, Figure 6A). The distribution peaked at around 20% of the cell cycle (i.e. around 4 hours after last division, Figure 6A). The onset of the second pulse was more widely distributed, which was approximately between S and G2/M phase (i.e. around 14 hours after last division, Figure 6B). Noticeably, most of the cells (198 out of 200) showed at least one p53 pulse during one cell cycle and a small fraction of cells (2 out of 200) displayed random fluctuations (Figure 6C and D, Figure S3). Most cells (61%) displayed 1∼2 pulses while 38% of total cells showed more than 2 pulses (Figure 6D and Figure S3). Therefore, the cumulative distribution plot in Figure 6C does not converge to 1 at 100% of the cell cycle. These results are either qualitatively or quantitatively consistent with Loewer *et al*'s measurements [7].

### Evaluation of the excitability of p53 pulses

**Figure 7:**
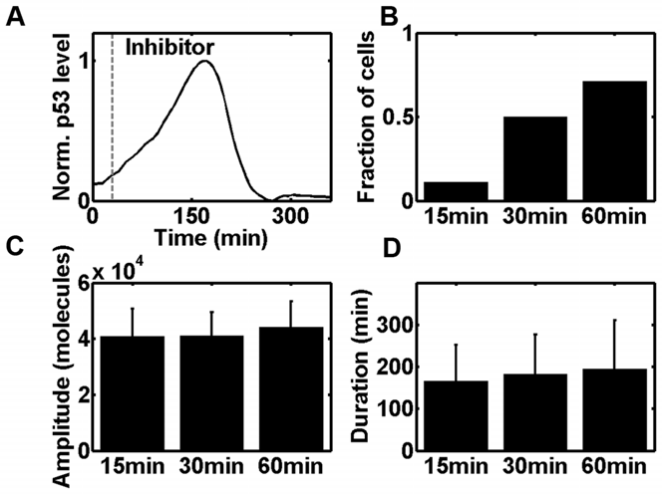
Excitability of p53 pulses. (A) A sample trajectory is shown where inhibitor was added after 30 min (dashed line: 30 min, set k_auto_ and k_DSB_ = 0 after inhibitor is added). (B) Fraction of cells showing a p53 pulse. (C and D) The amplitude of p53 pulses (C) and their duration (D) were unaltered (error bars indicate the standard deviation).

Next we examined whether a transient stimulus could induce a p53 pulse. We challenged the stochastic system with external DNA damage (set DSB = 300, i.e. approximately 10 Gy γ-irradiation [18]) similar to the experimental treatment with neocarzinostatin (NCS) [7]. We also set DSB = 600 and obtained qualitatively similar results (data not shown). Note that under such circumstances, the spontaneous induction of endogenous DSB was shuttered because strong DNA damage might invoke cell cycle arrest [19]. After a given time interval, we inhibited ataxia telangiectasia mutated (ATM) activation by setting all the activating coefficients to be 0 which is similar to the experimental treatment of cells with ATM kinase inhibitor Wortmannin (Wm) [7]. A fraction of trajectories showed that indeed p53 levels continue to increase after inhibition, resulting in a full p53 pulse (30 min before inhibition, Figure 7A). We further reduced the pre-inhibition time and found that the fraction of simulations that leads to a full pulse dramatically decreased (for 15min, 11%, Figure 7B). To the opposite, once the pre-inhibition time was elongated, the fraction increased (for 60 min, 71%, Figure 7B). The excited pulses did not vary significantly both in amplitude and duration (Figure 7C and 7D). These results suggested that p53 pulses are excitable. The excitability of p53 pulses in our model is consistent with experimental results [7].

### ATM mediated post translational modification determines effector molecule expression

**Figure 8:**
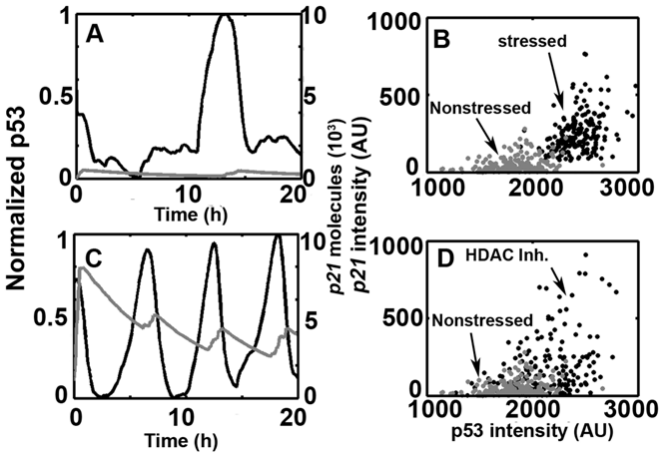
P53 and *p21* dynamics under stressed and nonstressed conditions. (A and C) *p21* (grey) and normalized p53 pulses (black). (B and D) Scatter plots of p53 and p21 integrated responses. (B) Stressed (black) versus nonstressed (grey) conditions. (D) HDAC (black) versus nonstressed (grey) conditions, HDAC: histone deacetylase.

Based on the experimental results (see materials and methods), we assumed that the fully competent form of p53 (P53_a_) is activated through ATM mediated activation (e.g. acetylation, [7]). Note that the first term in Eq.6 is not only restricted to ATM mediated acetylation but also other ATM mediated activating effects. We chose *p21* (P21mRNA) as the output as Loewer *et al.* [7]. Under nonstressed conditions, only spontaneous p53 pulses were triggered and *p21* did not show substantial expression (Figure 8A, *p21*: grey curve). In response to external DNA damage (i.e. set DSB = 300 in our model), however, the system showed regular and synchronous pulses and *p21* also exhibited significantly high expression (Figure 8C, *p21*: grey curve). We further evaluated the integrated responses of P53 and *p21* (i.e. the integration along the time, 200 samples) and found that the induction levels of *p21* under stressed and nonstressed conditions differ significantly (Figure 8B, compare black and grey dots). Noticeably, in Loewer *et al.*'s experiments, also shows sporadic high induction levels *p21* under nonstressed conditions (compare Figure 5F and 5G in [7]). Therefore, the overlay between black and grey dots in Figure 8B could be acceptable consistency. In order to investigate whether reduced inhibition of ATM mediated p53 full activation (i.e. P53_a_ production) could also lead to visibly high levels of *p21* in the absence of external DNA damage, we *in silico* decreased the deacetylation rate (i.e. set k_deact_ = 0.01, similar to the treatment with HDAC in Loewer et al's experiments [7]). As expected, we also observed substantial elevation of *p21* levels in comparison with unperturbed control (Figure 8D). However, there seems to be some slight deviation to experiment results (e.g. Figure S4 in [7]) and this issue will be discussed below.

## Discussion

Biological system is also confronted with stress of various sources. Of profound importance is DNA damage which sabotages the integrity of genome and living organism [20]–[21]. Meanwhile, a homeostatic system ought to be capable of discriminating internally spontaneous damage that is normally repairable and severe external damage that might lead to disastrous consequences. The p53 pathway presents a promising aspect on this issue as it can filter out transient, endogenous damage and induce substantial amount of effectors (e.g. *p21*) when cells have suffered tremendous stress. Therefore, theoretical modeling of basal p53 dynamics creates a fertile ground for understanding the homeostatic control of biological system.

Most studies focus on the generation of p53 pulses after stress. Little is known about the basal dynamics of p53 in proliferating cells. It was not until 2010 that Loewer et al's first published their preeminent work on unraveling basal p53 pulses under nonstressed conditions. However, no mathematical models have been constructed to investigate the basal p53 pulses since then. Meanwhile, as basal p53 pulses under nonstressed conditions characterize new features in p53 network (reminiscent of uncovering digital p53 oscillation in 2004), no earlier model can be directly applied. Also, some major points (e.g. reproduce basal p53 pulses, the excitable nature, the relation between first peaking times and cell cycle and more importantly, the physiological roles of basal pulses) proposed in Loewer et al's experiments have not been investigated using mathematical models. Therefore, we constructed a mathematical model to investigate these new features in p53 dynamics.

In current work, we presented a novel refinement to the TLK model and characterized some key properties of p53 basal pulses. We found that under basal, nonstressed conditions, p53 showed transient, irregular and spontaneous pulses (Figure 5 and Figures S2 and S3). The spontaneous pulses are highly variable and asynchronous (Figures 5B, 5C and S2). Meanwhile, the pulses showed significant correlation with cell cycle phase and a predominant G1 distribution in first pulsing time (Figure 6A and 6C). We further unraveled the excitable nature of p53 pulses *in silico* and demonstrated the stability of period and amplitude (Figure 7). Furthermore, we also gave qualitative credence to the supposition postulated by Loewer et al that posttranslational modifications dictate the cellular decision between spontaneous and sustained external damage (Figure 8 and Ref [7]).

A special attention should be paid to the terminology ‘nonstressed condition’. Nonstressed condition is exactly in the sense that cells are confronted with spontaneous and endogenous DNA breaks of various sources. Sister chromosome recombination, chromatin decondensation, thermodynamic fluctuation, oxidative species and enzymatic activities of telomerase all contributed to the background occurrence of DNA breaks [18], [22]. As the explicit pathways that lead to breaks production is elusive, we approximated the endogenous DSB production using a modified TLK model without resorting to the exact molecular pathways. Also we only considered the DSB production during cell cycle progression to establish a correlation between pulses and cell cycle phase. Incorporation of other sources presents a daunting challenge and remained to be evaluated in future.

Some other models also investigated the stochastic p53 pulses or the dynamics of p53 under ‘non-stressed’ conditions, respectively [23]–[24]. However, In Bottani et al's model, non-stressed condition refers to a state when no DNA damage exists (i.e. both extrinsic and intrinsic) [23]. The term ‘nonstressed condition’ in Loewer et al.'s experiments corresponds to the situation that intrinsic and physiological DNA damage produced during normal cell cycle progression does exist. Therefore, nonzero intrinsic DNA damage identified experimentally may challenge Bottani’s model. Cai et al. performed stochastic simulation in p53 system. However, their model is constructed to explore the variation of p53/MDM2 oscillations in Geva-Zatorsky et al.'s experiments [17]. Furthermore, Batchelor et al experimentally identified that both p53-MDM2 and p53-Wip1-ATM negative feedback loops are indispensable to give rise to sustained and uniform p53 pulses [6]. However, Neither Cai et al's nor Bottani et al's model incorporated the p53-Wip1-ATM negative feedback loop. Since the basal p53 dynamics has not been uncovered until Loewer et al.'s preeminent work is published, we argue that the stochastic simulation in our model may characterize new features in p53 dynamics.

In our refined TLK model, we found a fraction of cells harbor nonzero (low level) DNA breaks during the phase transition (i.e. S to G2 or G2/M to G1). How could that occur in normal cell cycle progression? Accumulating evidence has suggested that cancer cells have relaxed cell cycle control and can propagate unrepaired breaks through cell cycle progression with wild type p53 ([25] and references therein). Cells can also commit division even in the presence of double strand breaks and the rate of progression seems unaffected by the amount of DNA damage [25]–[29]. These results could validate our model assumption on the stable cell cycle progression.

Since the fact that (cancer) cells undergo cell cycle progression even in the presence of DNA damage has been identified, then why first peaks show a predominant G1 distribution (Figure 6A and 6C)? We hypothesized the unrepaired DNA breaks and/or residual activated ATM molecules that propagate to the daughter cells might be the trigger of p53 pulses. It has been suggested that mitosis could also lead to detectable DNA damage [22], [30]–[31]. Therefore, when a cell divides, the daughter cell that ‘inherits’ the DNA breaks might induce a p53 pulse through excitable mechanism. Even the DSB is fully repaired when division occurs, there might be also residual or considerable amount of phosphorylated ATM that could enter daughter cells. The excitable nature of p53 pulses (Figure 7 and [7]) guarantees that daughter cells may also trigger a full pulse with a certain probability. Possibly, only these cells that do not ‘receive’ parental DNA damage (and possibly activated ATM) and fail to initiate an excitable p53 pulse (with residual activated ATM) may continue to randomly fluctuate until new spontaneous DNA damage is encountered.

In Lower *et al.*'s experiments, they also observed that G2 arrested cells show strongly reduced number of p53 pulses [7]. How can it be interpreted using our model? In our model, G2 (or G2/M) is the other source of endogenous DSB production. Since cells are arrested in G2 phase, they will not undergo division. At earlier times of arrested G2 phase, spontaneous DSBs might invoke p53 pulses through excitable mechanism. DNA breaks are consecutively repaired. However, since cells are arrested, they cannot divide or enter next cycle, which means there will not be other spontaneous DSBs produced (Note that in our model, only S and G2 phases produce DSBs). Cells will eventually stop pulsing and perform random fluctuations. Therefore, the number of pulses is reduced by taking mean values compared with proliferative cells.

The observation that p53 can filter spontaneous pulses and remain sensitive to external DNA damage is reminiscent of coherent feed-forward loops as suggested by Loewer *et al.* [7], [32]. When spontaneous damage is encountered, p53 becomes accumulated and form asynchronous pulses through excitable mechanism. When external damage is severe and sustained, substantially activating modifications (e.g. acetylation) will be accomplished that finally lead to full *p21* activation (Figure 8 and Ref [7]). Noticeably, p21 is positioned as a master effector of multiple anti-proliferative pathways [33]. P21 inhibits CDK activities of broad classes, restrains the expression of genes that are critical for cell cycle progression by direct binding (e.g. E2F1, Myc, STAT3), curtails DNA synthesis through binding to proliferative cell nuclear antigen (PCNA) and suppresses apoptosis by inhibiting the activities of procaspase 3, caspase 8, caspase 10 and stress-activated protein kinases (SAPKs) [34]–[37]. Therefore, the regulatory mechanisms of p21 may not restrict itself to G1 arrest but can be extended and contribute to a large cohort of physiological processes. Taken together, the tolerant and sensitive nature of p53 pulses is crucially important for homeostasis control.

We should note that simplified models cannot cover all the characterized interaction (e.g. Chk1 is not included in our model as discussed in materials and methods). It was recently reported that MDM2 regulates p53 translation [38]. Recent experiments also identified a plausible positive feedback loop between p21 and p53 [39]. The latter finding should be interpreted with caution as they only established correlations between corresponding protein levels. Meanwhile, p53 system is pervaded with feedback loops although they might be stress and context dependent [40]. As suggest by Lahav and coworkers, we only incorporated two negative feedback loops and associated time delays in our model [41]. We did not investigate all the ATM mediated posttranslationally activating effects for simplification (only taking acetylation as a case study). From another point, since the spontaneous DSB production pathway is ambiguous, we only modified the TLK model to model this process. Although not explicitly or precisely characterized (e.g. step size and updating probabilities), our model is at least partially consistent with experimental results. Noticeably, there seems to be some deviation between model fit and experiments (e.g. Figure 6 and 7). A critical issue argues that some cells might stop dividing while our model assumption states that all cells will eventually divide. This consideration might add another layer of complexity but cannot be feasibly incorporated into the model. Since considerable stochasticity exists in real biological system, the stochasticity incorporated in our model only denotes parts of all and stochasticity control cannot be easily manipulated [42]. Meanwhile, in Loewer et al's experiments, the *p21* seems to undergo a perfectly stepwise elevation. However, the degradation rate of *p21* is reported around 5.5 hours ([43] i.e. ∼0.0021 µM•min^−1^ and we take 0.002 µM•min^−1^ in our model). The stepwise elevation requires no or extremely low degradation [44]. It is not demonstrated whether the fluorescence tagged *p21* lowers the degradation which ultimately engenders a perfectly stepwise elevation. At least, these results are qualitatively consistent with experiments. As more details come in torrents, the model can be refined in future.

Our model characterized the basal dynamics of p53 pulses and identified both the tolerant and sensitive nature of p53 network. The intricate regulation of p53 network may also exert a global control over homeostasis by interactions with other signaling network such as NF-κB [45]. It will be important to investigate these interactions and uncover the hidden layer of complexity.

## Materials and Methods

### Model construction

The model consists of 12 species and 31 reactions. For p53, MDM2 p21 and WIP1, we incorporated both mRNA and protein species. Although p21mRNA (*p21*) was selected as the model output which is accordant with Loewer *et al.*, the p21 protein is added for symmetric purpose. Total ATM was set a constant because experiments showed that the levels of ATM are relatively stable within 72 hours [46]. Double strand breaks can directly activate ATM [47]. Meanwhile, ATM is also activated in response to DNA damage through intermolecular autophosphorylation [48]. Activated ATM (i.e. phosphorylated form) then phosphorylates and stabilizes p53 (p53_p_) [49]. ATM can also phosphorylate MDM2 and destabilize it [50]–[51]. Under nonstressed conditions, MDM2 is relatively stable and targets p53 for degradation [51]. However, under stressed conditions, activated ATM can phosphorylate and destabilize MDM2 [51]. The ability to degrade p53 is strongly diminished for phosphorylated MDM2 [5], [52]. According to Leower et al., we assumed that ATM can also induce p53's acetylation indirectly via intermediate enzymes [7]. A further assumption was made that both modified p53 species (P53_p_ and P53_a_) can activate *mdm2* and *wip1* transcription [53] and only the fully competent form (P53_a_) can induce *p21* [7]. Noticeably, we also split the third term in Eq.3 (also Eq.4) into two separated transcription terms and found quantitatively similar dynamics (data not shown). The activating coefficient (k_atm3_) in the first term (Eq.6) is rescaled to exert dynamic control (for the same reason, k_DSB_ was rescaled). Note that the simplified first term (Eq.6) should be interpreted with caution as it not only describes ATM mediated acetylation but also other ATM mediated activating effects. We further assumed that deacetylated p53_a_ is deprived of full competence which can only activate *mdm2* and *wip1* transcription (i.e. an equivalent form of p53_p_). Recent experiments showed that WIP1 can dephosphorylate Chk2, activated ATM, and phosphorylated forms of both p53 and MDM2. We did not incorporate Chk2 in our model for simplification without compromising the input-output relation because ATM can bypass Chk2 and directly activate p53 [54]. WIP1 mediated Chk2 dephosphorylation can be envisioned as a direct (partial) inhibition of ATM induced p53 activation. All the kinetic interactions are schematically described in Figure 1. We formulated these interactions by ordinary differential equations (ODE) (Table S1). The parameters and their biological descriptions are shown in Table S2.

### Stochastic simulation

#### DNA damage repair module

The cell cycles of transformed cells used in experiments are approximately 20 hours and relatively stable [7], [17]. Meanwhile, according to Loewer *et al.*'s measurement, the ratio of the time spent in each cell cycle phase approaches a constant 3∶4∶3 (see supplemental materials in Ref [7]). The phase between S and G1 is referred to as G2/M phase [22]. Noticeably, the DNA content immunofluorescence experiments (e.g. Figure S3C in Ref [7]) do not distinguish the G2/M boundary. Furthermore, the M phase duration is very short compared with interphase [55] and we also assumed an instant cell division (see next section). For these reasons, we incorporated G2 and M into a single phase G2/M which is quantitatively the same as G2 phase in Ref [7]. We did not incorporate an explicit cell cycle model with p53 oscillator for two reasons. Firstly, we aim to investigate the basal p53 pulses. Sophisticated cell cycle model is far too complex and might sabotage stochastic simulations making evaluating p53 pulses infeasible. Secondly and more importantly, although cell cycle models are well established, the explicit signaling pathways linking endogenous DSB production and cell cycle progression (S and G2/M phase, see below) is ambiguous and casts a cloud on model formulation. Because DNA repair module serves as only input to downstream oscillator and the cell cycle length is relatively stable, we approximated one cell cycle by 20 hour with phase length ratio 3∶4∶3 (i.e. G1: 6 hours, S: 8 hours, G2/M: 6 hours). The major source of endogenous double strand breaks (DSB) comes from DNA replication (e.g. single strand lesions conversion to DSB) and mitosis (e.g. chromosome decondensation) and during which S phase plays a major role [18], [22], [31]. The spontaneous production rate of EDSBs is approximately 50 per cell per cycle [18]. We assume that 40 DSBs are produced during S phase and 10 DSBs during G2/M phase (45: 5 is also feasible and we only performed a case study). The 40 DSBs are randomly produced during S phase (i.e. 7–14 hours in one cell cycle) with uniformly distributed producing time. The 10 DSBs in G2/M phase (i.e. the 15–20 hour during one cell cycle) follow similar procedure with uniformly distributed times. Then the algorithm proceeded by maintaining a sequence structure of DSB producing time. The sequence was checked in each iteration to determine whether a new DSB was produced or not. If it was indeed the case, the newly produced spontaneous DSBs were added to the total amount of unrepaired DSB. The treatment of DSB is reminiscent of that of τ leap method. For DSB repair process, we adopted a two-lesion-kinetics (TLK) model developed by Ma *et al.* [15] (see Figure S5A). For details, please refer to Ref [15]. In Ma *et al.*'s model, based on experimental results, it is assumed that 70% of the total DSB is processed by fast repair and 30% by slow repair. Therefore, we modified the model as follows: For each spontaneous DSB, it will be repaired by fast kinetics with a probability of 0.7, and by slow kinetics with a probability of 0.3. To implement step size control, we chose a relatively small step size (Δt = 0.2, which is smaller than the shortest τ in τ-leap method in our simulation). We updated the DNA repair module by consecutive Δt (i.e. n•Δt, where n = τ mod Δt, ‘mod’ denotes modulus after division, Figure S5B). To retain compatibility with τ-leap size, the last step size was set to be τ-n•Δt. Therefore, the evolution of DNA repair module can accommodate downstream oscillator (For a brief introduction of our modified TLK model, please refer to Text S1). The number of unrepaired DSBs can activate ATM and function as the input to downstream module. Note that p53 was reported either to suppress or to promote DNA damage repair [56]. Therefore, we followed the formulation by Ma et al and did not modify the fixation rates by p53 related terms [15]. A typical run of the DNA damage module is represented in Figure S4 (upper panels).

#### Oscillator module

As most of the molecular species are of the order of 10^3^∼10^5^, we formulated the stochastic ODE system using binomial delay τ-leap method according to Chatterjee *et al* and Leier *et al.* [57]–[58]. All the delays were varied by 30% from reference values. Furthermore, since growing evidence has confirmed quantal mRNA transcription and increasing the level of transcription factors increases the average size of the bursts [59]. We modeled all the transcription reactions as transcriptional burst following Golding *et al.* [60]. We further assumed that there are two gene copies and both copies are stochastically shifted between ON and OFF state following Puszyński *et al.* (i.e. the transcription reactions are multiplied by a random variable *G*, where *G = G1+G2*, *G1* and *G2* either take 0 or 1) [61]. Codes are available as Text S2.

### Synchronization of cell cycle

Noticeably, the dynamics of p53 is quantified in transformed cell lines (e.g. MCF7, HCT116) but not primary cells. The transformed cells have undergone many generations (i.e. have divided several times). To approximate the real dynamics, we primed cells for one generation (i.e. ran the stochastic simulation for one cell cycle, in our model, 20 hours), continued the simulation when cell division occurred, but rescaled the time (Figure S6, i.e. when cells divides, the time is reset to 0 h). We assumed that cell undergoes an instant division and all the molecules were binomially distributed towards sister cells [60], [62]. We recorded the trajectories after the rescaling (total 200 samples, from rescaled 0 to 20 hours, Figure S6). Each trajectory is then normalized according to Geva-Zatorsky *et al.* [17]. After this procedure, the cell cycle was synchronized. In Loewer *et al*'s experiments, they also synchronize the cell cycle to investigate the basal p53 pulses [7]. Therefore, our treatment is well consistent with experiments.

### Total parameter variation (TPV)

A relative (normalized and absolute) change for an output *Y* is defined as follows:
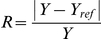
Here *Y* is a typical output (i.e. period and amplitude) in one stochastic parameter set, while *Y_ref_* is the nominal model output in the reference parameter set. We plot for each parameter set the calculated relative change (*R*) as a function of the total parameter variation. TPV is defined as the total order of magnitude of parameter variation [63]:
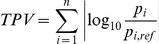
Here *n* is the number of the parameters in the model and *p_i_* and *p_i,ref_* are parameters in one stochastic set and reference parameter set respectively. Latin hypercube sampling (LHS) was used in our simulations. 1000 random parameter sets were generated using MATLAB built-in function *lhsdesign*. TPV can be envisioned as a measure for distance in kinetic parameter sets.

### Bifurcation analysis and model simulation

The deterministic delayed differential equations were integrated using dde23 solver in MATLAB. Bifurcation analysis of DDE was implemented with DDE-BIFTOOL, v. 2.00, a MATLAB package for bifurcation analysis of delay differential equations [64]. All simulations were carried out using MATLAB (MathWork, Version 7, Release 14).

## Supporting Information

Figure S1 Bifurcation diagram. Stable steady state (black solid curve), unstable steady state (dashed curve) and amplitude (violet) are shown. Red curve is a guide for the parameter value used in our model.
(TIF)

Figure S2 Distribution of the interspike intervals displays significant variations. The total number of stochastic runs is 200. However, a fraction of cells does not show pulses or only shows one single pulse (our time of interest is one cell cycle, i.e. 20 hours and therefore the intervals do not exceed 20 hours).
(TIF)

Figure S3 Representative dynamics of p53 under nonstressed conditions.
(TIF)

Figure S4 Representative dynamics of DSB repair process and associated dynamics of p53 and *p21*. DSB (violet), p53 (black) and *p21* (grey).
(TIF)

Figure S5 Schematic representation of DSB repair module and step size control. Δt: step size in DSB repair module. τ: step size in τ-leap method. (Note that even the smallest τ is longer than Δt = 0.01). Black dots denote spontaneous DSBs.
(TIF)

Figure S6 The time rescaling process during cell cycle synchronization. Dashed line indicates cell division.
(TIF)

Table S1 Ordinary differential equations for the model.
(DOC)

Table S2 Model parameters and description.
(DOC)

Text S1 A brief introduction to the modified TLK algorithm.
(DOC)

Text S2 The source code to generate the p53 basal pulses.
(M) Click here for additional data file.
