# Supplementary figures and images for: Modeling the Basal Dynamics of P53 System
Source: PLoS One. 2011 Nov 16;6(11):e27882. doi: 10.1371/journal.pone.0027882 (PMC3218058; doi:10.1371/journal.pone.0027882)

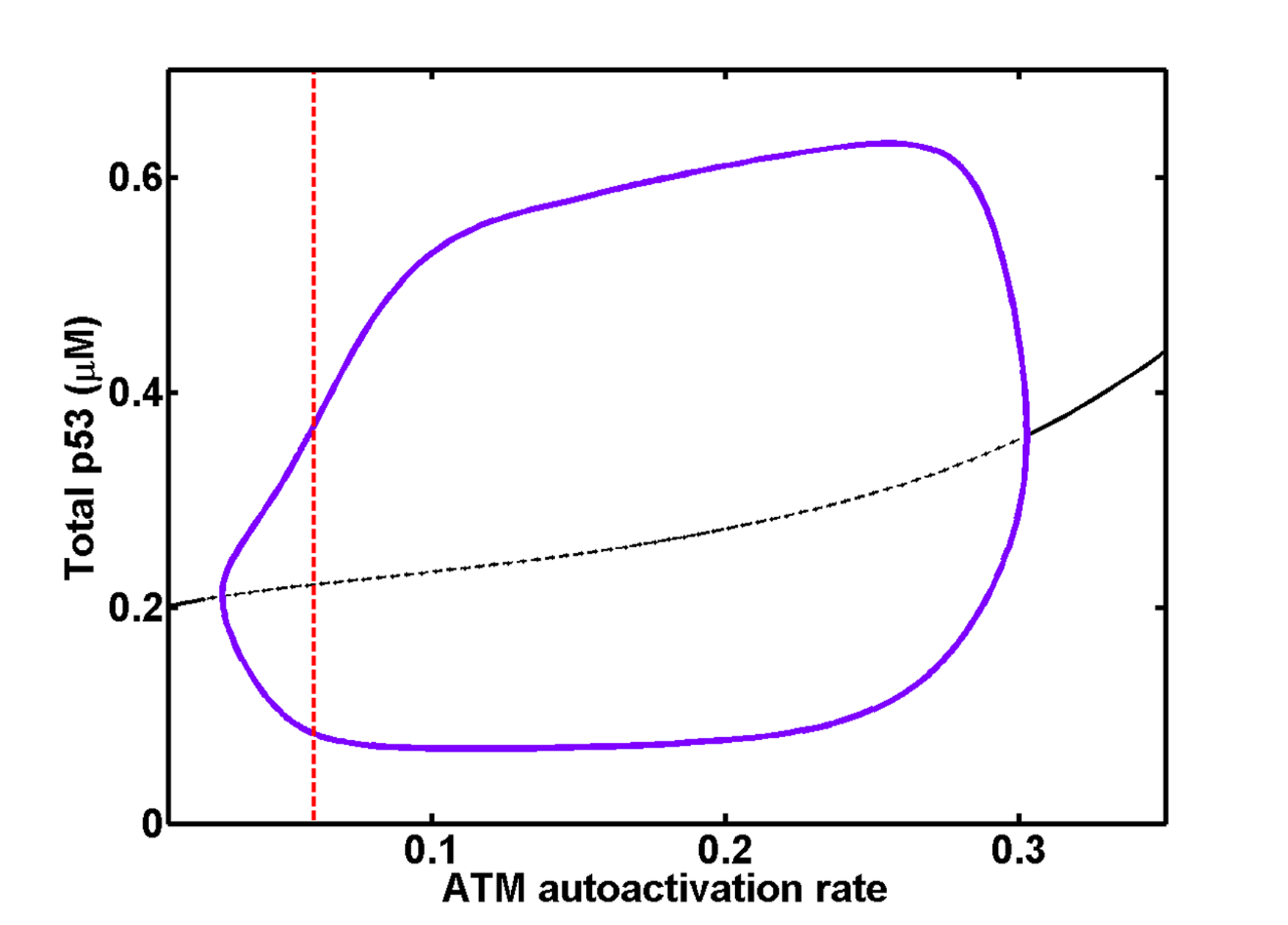

Supplement: Figure S1 — Bifurcation diagram. Stable steady state (black solid curve), unstable steady state (dashed curve) and amplitude (violet) are shown. Red curve is a guide for the parameter value used in our model. (TIF) [file pone.0027882.s001.tif]

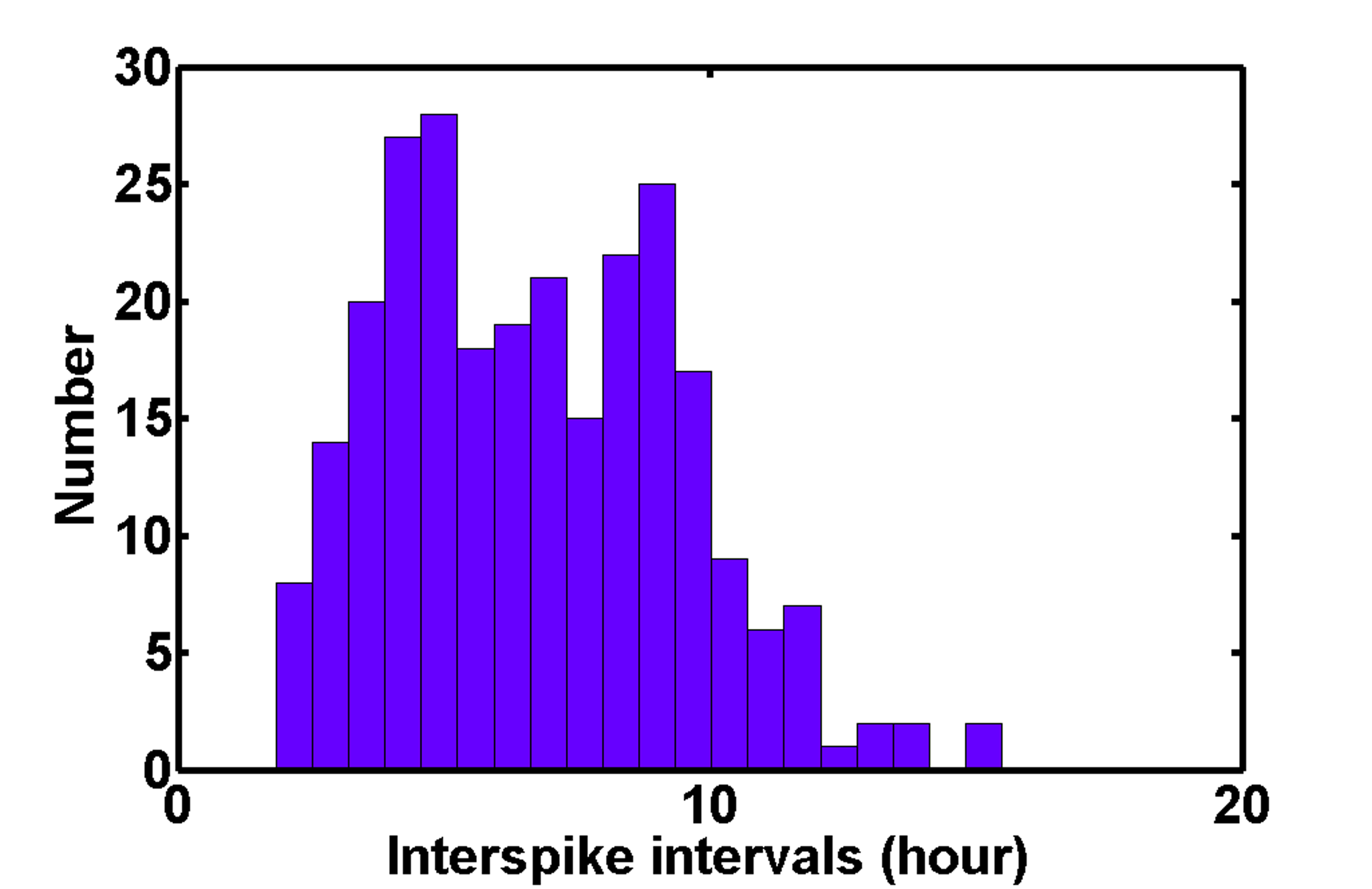

Supplement: Figure S2 — Distribution of the interspike intervals displays significant variations. The total number of stochastic runs is 200. However, a fraction of cells does not show pulses or only shows one single pulse (our time of interest is one cell cycle, i.e. 20 hours and therefore the intervals do not exceed 20 hours). (TIF) [file pone.0027882.s002.tif]

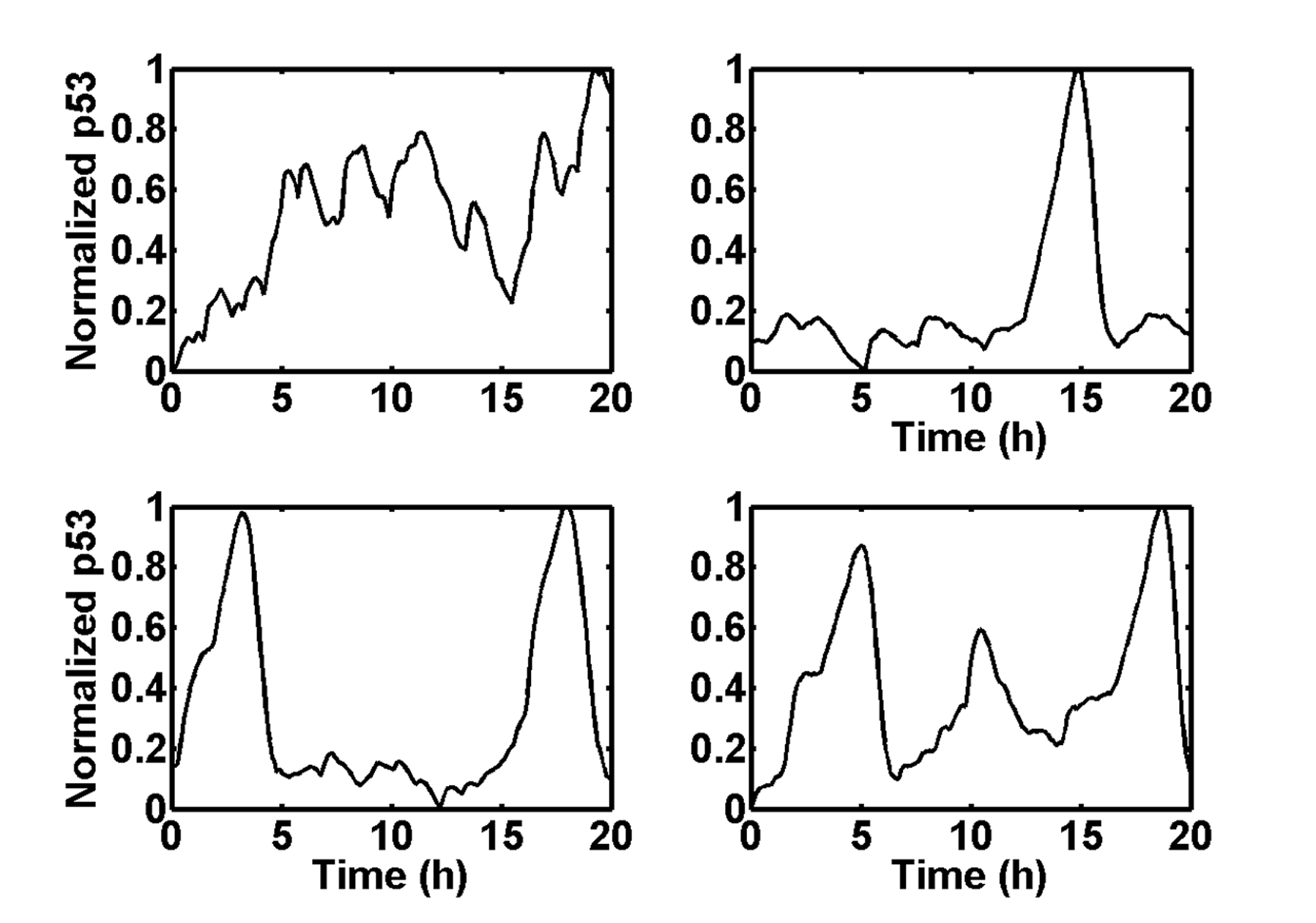

Supplement: Figure S3 — Representative dynamics of p53 under nonstressed conditions. (TIF) [file pone.0027882.s003.tif]

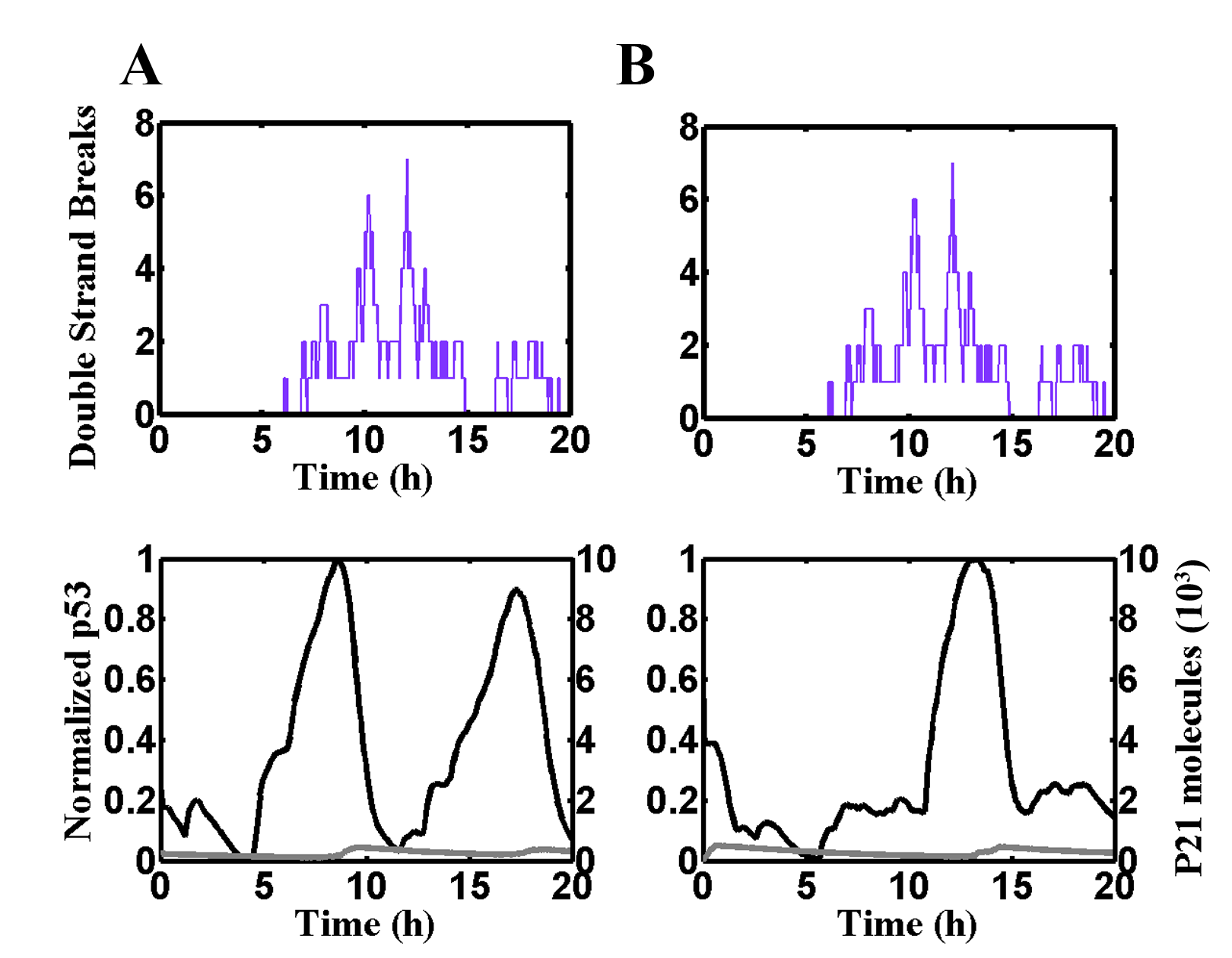

Supplement: Figure S4 — Representative dynamics of DSB repair process and associated dynamics of p53 and p21. DSB (violet), p53 (black) and p21 (grey). (TIF) [file pone.0027882.s004.tif]

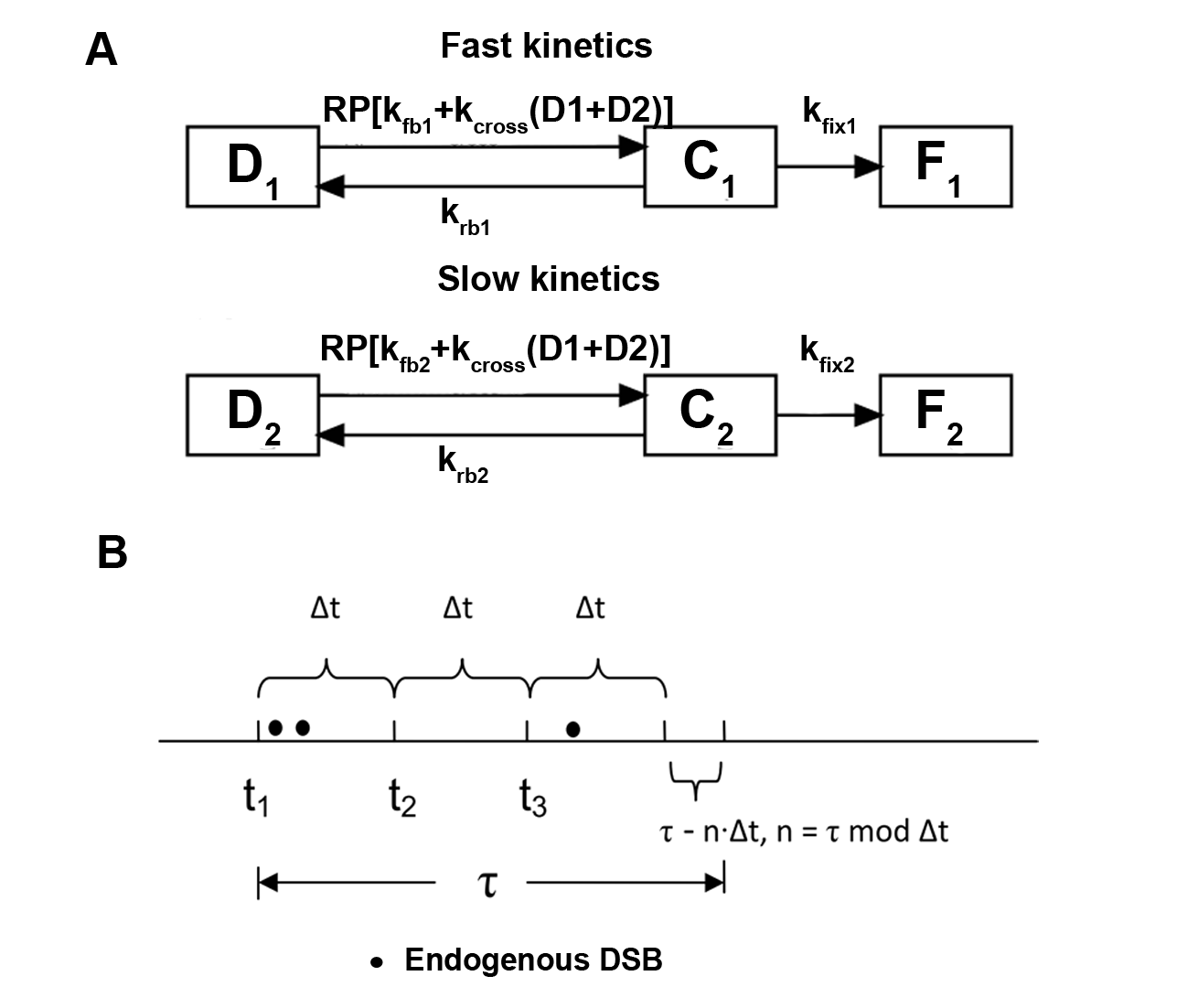

Supplement: Figure S5 — Schematic representation of DSB repair module and step size control. Δt: step size in DSB repair module. τ: step size in τ-leap method. (Note that even the smallest τ is longer than Δt = 0.01). Black dots denote spontaneous DSBs. (TIF) [file pone.0027882.s005.tif]

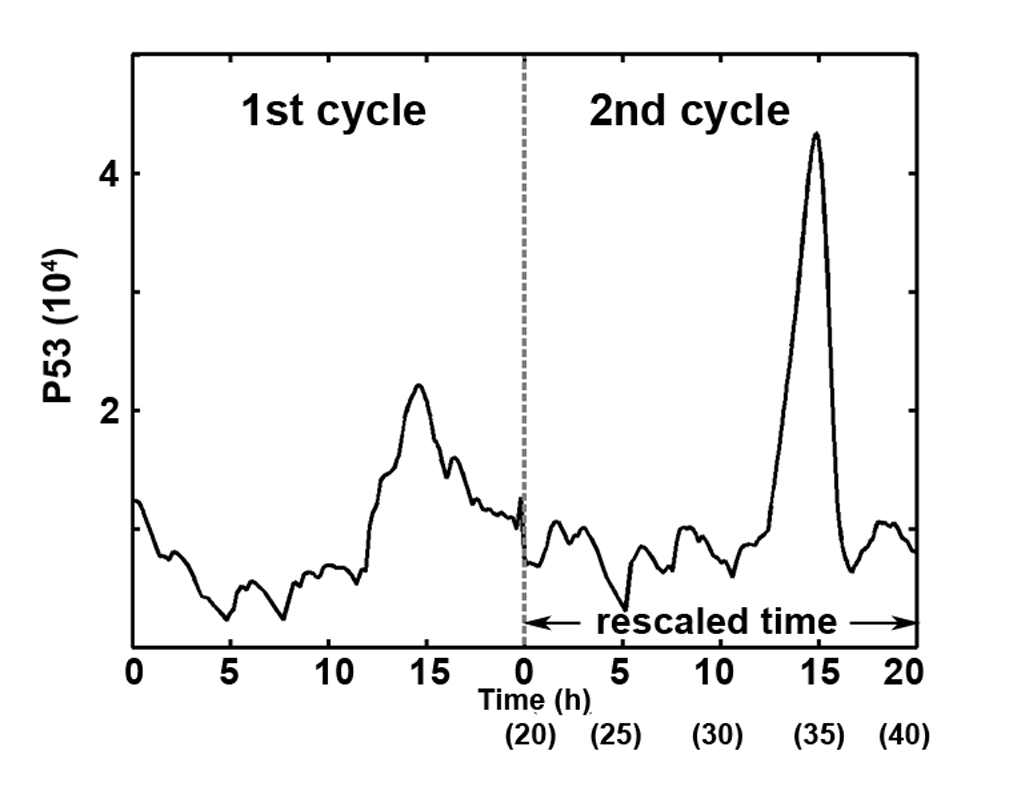

Supplement: Figure S6 — The time rescaling process during cell cycle synchronization. Dashed line indicates cell division. (TIF) [file pone.0027882.s006.tif]
