# Supplementary material for: Modeling the Basal Dynamics of P53 System
Source: PLoS One. 2011 Nov 16;6(11):e27882. doi: 10.1371/journal.pone.0027882 (PMC3218058; doi:10.1371/journal.pone.0027882)
Supplement: Table S1 — Ordinary differential equations for the model. (DOC) [file pone.0027882.s007.doc]

| $\frac{dp53}{dt}=s_{p53}-\delta_{p53}\cdot p53$ | (1) |
| --- | --- |
| $\frac{dmdm2}{dt}=s_{mdm2}-\delta_{mdm2}\cdot mdm2+e_{1}\cdot\frac{(P53p(t-\tau_{1})+P53a(t-\tau_{1}))^{4}}{(P53p(t-\tau_{1})+P53a(t-\tau_{1}))^{4}+K_{m}^{4}}$ | (2) |
| $\frac{dwip1}{dt}=s_{wip1}-\delta_{wip1}\cdot wip1+e_{2}\cdot\frac{(P53p(t-\tau_{2})+P53a(t-\tau_{2}))^{4}}{(P53p(t-\tau_{2})+P53a(t-\tau_{2}))^{4}+K_{w}^{4}}$ | (3) |
| $\begin{matrix} \frac{dP53}{dt}=r_{p53}\cdot p53-\mu_{P53}\cdot P53-k_{1}\cdot MDM2\cdot P53 \\ -k_{atm1}\cdot ATM*\cdot P53+k_{WIP1}\cdot P53p\cdot WIP1 \end{matrix}$ | (4) |
| $\begin{matrix} \frac{dP53p}{dt}=k_{atm1}\cdot ATM*\cdot P53-k_{WIP1}\cdot P53p\cdot WIP1-k_{2}\cdot MDM2\cdot P53p \\ -k_{atm3}\cdot P53p+k_{deact}\cdot P53a \end{matrix}$ | (5) |
| $\frac{dP53a}{dt}=k_{atm3}\cdot P53p-k_{deact}\cdot P53a$ $\begin{matrix} \frac{dMDM2}{dt}=r_{mdm2}\cdot mdm2(t-\tau_{4})-\mu_{MDM2}\cdot MDM2 \\ -k_{atm2}\cdot MDM2\cdot ATM*+k_{WIP1}\cdot MDM2p\cdot WIP1 \end{matrix}$ | (6)  (7) |
| $\frac{dMDM2p}{dt}=k_{atm2}\cdot MDM2\cdot ATM*-k_{WIP1}\cdot MDM2p\cdot WIP1-\mu_{MDM2p}\cdot MDM2p$ | (8) |
| $\frac{dWIP1}{dt}=r_{wip1}\cdot wip1(t-\tau_{5})-\mu_{WIP1}\cdot WIP1$ | (9) |
| $\begin{matrix} \frac{dATM*}{dt}=k_{DSB}\cdot ATM+k_{auto}\cdot ATM*\cdot ATM \\ -k_{WIP2}\cdot WIP1\cdot ATM*-k_{basal}\cdot ATM* \end{matrix}$ | (10) |
| $\frac{dp21}{dt}=e_{3}\cdot\frac{P53a(t-\tau_{3})^{4}}{P53a(t-\tau_{3})^{4}+K_{p}^{4}}-\delta_{p21}\cdot p21$ $\frac{dP21}{dt}=r_{P21}\cdot p21(t-\tau_{6})-\mu_{P21}\cdot P21$ $ATM+ATM*=1\mu M$,$k_{atm3}=k_{atm3'}\cdot\frac{ATM*}{ATM*+K_{p}}$, $k_{DSB}=k_{DSB'}\cdot\frac{DSB}{DSB+K_{DSB}}$ | (11)  (12) |

**Table S1:** **Ordinary differential equations for the model.**

Letters in lowercase and italics (*p53, mdm2, wip1* and *p21*) denote mRNA.
