# Supplementary material for: Modeling the Basal Dynamics of P53 System
Source: PLoS One. 2011 Nov 16;6(11):e27882. doi: 10.1371/journal.pone.0027882 (PMC3218058; doi:10.1371/journal.pone.0027882)
Supplement: Table S2 — Model parameters and description. (DOC) [file pone.0027882.s008.doc]

**Table S2: Model parameters and description.**

| **Parameters** | **Description** | **Value^a^** |
| --- | --- | --- |
| [*p53*]_0_ | Initial concentration P53 mRNA | 0.05 |
| [*mdm2*]_0_ | Initial concentration of MDM2 mRNA | 0.05 |
| [*wip1*]_0_ | Initial concentration of WIP1 mRNA | 0.04 |
| [P53]_0_ | Initial concentration of P53 | 0.1228 |
| [MDM2]_0_ | Initial concentration of MDM2 | 0.0286 |
| [WIP1]_0_ | Initial concentration of WIP1 | 0.0178 |
| [ATM] | Unphosphorylated ATM | 1 |
| s_p53_ | Production rate of P53 mRNA | 0.0005 |
| s_mdm2_ | Production rate of MDM2 mRNA | 0.001 |
| s_wip1_ | Production rate of WIP1 mRNA | 0.002 |
| δ_p53_ | Degradation rate of P53 mRNA | 0.01 |
| δ_mdm2_ | Degradation rate of MDM2 mRNA | 0.02 |
| δ_wip1_ | Degradation rate of WIP1 mRNA | 0.05 |
| δ_p21_ | Degradation rate of P21 mRNA | 0.002 |
| r_p53_ | Translation rate of P53 | 0.1 |
| r_mdm2_ | Translation rate of MDM2 | 0.02 |
| r_wip1_ | Translation rate of WIP1 | 0.02 |
| r_p21_ | Translation rate of P21 | 0.01 |
| μ_P53_ | Degradation rate of P53 | 0.035 |
| μ_MDM2_ | Degradation rate of MDM2 | 0.035 |
| μ_MDM2p_ | Degradation rate of MDM2p | 0.14 |
| μ_WIP1_ | Degradation rate of WIP1 | 0.035 |
| μ_P21_ | Degradation rate of P21 | 0.003 |
| e_1_ | P53 dependent *mdm2* transcription rate | 0.014 |
| e_2_ | P53 dependent *wip1* transcription rate | 0.014 |
| e_3_ | P53 dependent *p21* transcription rate | 0.2 |
| k_1_ | MDM2 dependent P53 degradation | 0.2 |
| k_2_ | MDM2 dependent P53p degradation | 0.01 |
| k_atm1_ | ATM* induced P53 phosphorylation | 1.8 |
| k_atm2_ | ATM* induced MDM2 phosphorylation | 0.01 |
| k_atm3’_ | ATM* mediated P53 acetylation | 0.3 |
| k_deact_ | P53a deacetylation | 0.05 |
| k_wip1_ | WIP1 induced P53p dephosphorylation | 1.5 |
| k_wip2_ | MDM2p dephosphorylation | 0.5 |
| k_DSB’_ | DSB induced ATM activation rate | 0.0005 |
| k_auto_ | ATM autoactivation rate | 0.06 |
| k_wip4_ | WIP1 induced ATM* dephosphorylation | 1.5 |
| k_basal_ | ATM* basal inactivation rate | 0.02 |
| K_m_ | EC_50_ for *mdm2* induced transcription | 0.2 |
| K_w_ | EC_50_ for *wip1* induced transcription | 0.2 |
| K_p_ | Threshold concentration | 0.01 |
| K_DSB_ | Activation scaling parameter | 200 |
| τ_1_ | *mdm2* transcriptional delay | 30 |
| τ_2_ | *wip1* transcriptional delay | 30 |
| τ_3_ | *p21* transcriptional delay | 30 |
| τ_4_ | MDM2 translation delay | 10 |
| τ_5_ | WIP1 translation delay | 10 |
| τ_6_ | P21 translation delay | 10 |
| k_fb1_ | association rate | 1.5 |
| k_cross_ | cross association rate | 0.01 |
| k_fix1_ | DSB fixation rate | 0.1 |
| k_rb1_ | dissociation rate | 0.1 |
| f_scale_ | scale parameter | 7 |

**a:** The first and second order rate constants are expressed in units of min^-1^ and μM^-1^∙ min^-1^, respectively. The production rates are expressed in units of μM∙ min^-1^. All delays take the unit min. The parameters in DSB repair module are in units of 1. The remaining parameters k_fb2_, k_fix2_, and k_rb2_ can be obtained through dividing by the scaling parameter. For detailed parameter information, please refer to the original reference.
