# Supplementary material for: Modeling the Basal Dynamics of P53 System
Source: PLoS One. 2011 Nov 16;6(11):e27882. doi: 10.1371/journal.pone.0027882 (PMC3218058; doi:10.1371/journal.pone.0027882)
Supplement: Text S1 — A brief introduction to the modified TLK algorithm. (DOC) [file pone.0027882.s009.doc]

**The implementation of stochastic module on basal DSB repair**

In this supplemental text, we make a brief introduction to our model about DNA double strand break (DSB) repair. This module is a direct modification from the Ma et al’s TLK model. According to Ma et al, there exist two repair pathways (i.e. fast and slow) [1]. Ma et al assumed that 70% of the total DSBs are repaired by fast kinetics, while remaining breaks are repaired through slow kinetics. Noticeably, Ma et al’s TLK model focuses on p53 digital oscillations under stressed conditions. That means, a nonzero DSB number is assigned as the initial value for repair if cells are irradiated with a certain dose of γ-rays. For example, if initial DSB is 100, then 70% (70 DSBs) of the total DSBs are repaired by fast kinetics, and 30% (30 DSBs) through slow kinetics. However, our model mainly focuses on basal p53 dynamics under nonstressed conditions. Under nonstressed conditions, the DSBs are not confronted when the simulation is initiated (i.e. the initial number of DSBs are zero, the start of G1 phase of cell cycle). During cell cycle progression, cells spontaneously produce DSBs through multiple mechanisms (see main text for details). Therefore, DSBs are encountered when intrinsic DNA damage occurs. As MCF7 has a relatively robust cell cycle time (20 hours), we approximated one cell cycle by 20 hour with phase length ratio 3:4:3 (i.e. G1: 6 hours, S: 8 hours, G2/M: 6 hours) [2]. We assumed there are 40 DSBs in S phase and 10 DSBs in G2/M phase of cell cycle (for details, see main text). The exact time for the occurrence of DSB is uniformed distributed within corresponding cell cycle phase (e.g. 40 random numbers with uniform distributions are generated within 7-14 hour). We modified the model as follows: for each spontaneous and intrinsic DSB, it will be repaired by fast kinetics with a probability of 0.7, and by slow kinetics with a probability of 0.3.

Given the small number of DSBs is spontaneously generated and the relatively small number of repair proteins, the DNA repair process was implemented through stochastic simulation. In DSB repair module, each locus in which a DSB is created can be in one of three states corresponding to intact DSB (state 1), DSB in complex with repair proteins (state 2), and (correctly or incorrectly) fixed DSB (state 3). At time step k, the number of DSBs in corresponding states (i.e. 1, 2 or 3) are represented by D(k), C(k), and F(k), respectively. By using subscripts ‘1’ and ‘2’ to differentiate simple DSBs (fast kinetics) and complex DSBs (slow kinetics), we have D(k) = D_1_(k) + D_2_(k), C(k) = C_1_(k) + C_2_(k) and F(k) = F_1_(k) + F_2_(k). The total number of repair proteins (RP) is assumed to be 20. At any time, variable RPs out of total 20 ones are free to bind to DSBs. To implement step size control, we chose a relatively small step size (Δt=0.2, which is smaller than the shortest τ in τ-leap method in our simulation). We updated the DNA repair module by consecutive Δt (i.e. n∙Δt, where n=τ mod Δt, ‘mod’ denotes modulus after division, Figure S4B). To retain compatibility with τ-leap size, the last step size was set to be τ-n∙Δt. The Monte Carlo algorithm for the evolution of DSBs during time [0, *tfinal*] is as follows:

1. Set the initial conditions. Set t=0. We set D_1_(0) = D_2_(0) =0, C_1_(0) = C_2_(0) =0 and F_1_(0) = F_2_(0) = 0. Given that initially all repair proteins are free, the simulation starts with total RP=20. There is no DSB until intrinsic DSBs are encountered. If a new DSB occurs, we assume that it is in state 1 (i.e. intact DSB). Set k=0 (i.e. storing time step).

2. Increment time. Set t=t+∆t (if not specifically indicated, see text above). Set k=k+1. If a DSB is generated during this time interval [t, t+∆t], D_1_(k)= D_1_(k)+1 (with a probability 0.7, fast kinetics) or D_2_(k)= D_2_(k)+1 (with a probability 0.3, slow kinetics).

3. Update the states for each of the damage sites controlled by fast repair. Compute the transition probabilities as follows:

From state 1 to state 2,

P_D1->C1_=RP[k_fb1_+k_cross_(D_1_(k-1)+D_2_(k-1))]∆t.

From state 2 to state 1,

P_C1->D1_=k_rb1_∆t.

From state 2 to state 3,

P_C1->F1_=k_fix1_∆t.

For each DSB, generate a value *x* from a uniform distribution with 0 and 1. If the DSB is in state 1, a transition to state 2 occurs if 0≤X<P_D1->C1_, while it stays in state 1 if P_D1->C1_≤X≤1. If the damage is in state 2, a transition to state 1 occurs if 0≤X<P_C1->D1_, or a transition to state 3 occurs if P_C1->D1_≤X<P_C1->D1_+ P_C1->F1_, or no transition occurs if P_C1->D1_+ P_C1->F1_≤X≤1. If the damage is in state 3, it stays in state 3 (i.e., state 3 is absorbing). Set RP=RP-1 if transition from state 1 to state 2 occurs; set RP = RP + 1 if transition from state 2 to state 1 occurs; otherwise RP remains the same. Count the number of fast repaired breaks at time t in states 1, 2, and 3 to be D*_1_*(k), C_1_(k), and F_1_(k), respectively.

4. Update the states for each of the damage sites controlled by fast repair. Compute the transition probabilities as follows:

From state 1 to state 2,

P_D2->C2_=RP[k_fb2_+k_cross_(D_1_(k-1)+D_2_(k-1))]∆t.

From state 2 to state 1,

P_C2->D2_=k_rb2_∆t.

From state 2 to state 3,

P_C2->F2_=k_fix2_∆t.

For each DSB, generate a value *x* from a uniform distribution with 0 and 1. If the damage in state 1, a transition to state 2 occurs if 0 ≤X<P_D2->C2_, while it stays in state 1 if P_D2->C2_≤X≤1. If the damage is in state 2, a transition to state 1 occurs if 0≤X<P_C2->D2_, or a transition to state 3 occurs if P_C2->D2_≤X<P_C2->D2_+ P_C2->F2_, or no transition occurs if P_C2->D2_+ P_C2->F2_≤X≤1. If the damage is in state 3, it stays in state 3 (i.e., state 3 is absorbing). Set RP = RP-1 if transition from state 1 to state 2 occurs; set RP = RP + 1 if transition from state 2 to state 1 occurs; otherwise RP remains the same. Count the number of fast repaired breaks at time t in states 1, 2, and 3 to be D_2_(k), C_2_(k), and F_2_(k), respectively.

5. Let D(k) = D_1_(k) + D_2_(k), C(k) =C_1_(k) + C_2_(k), and F(k) =F_1_(k) + F_2_(k).

6. Repeat steps 2-5 until t=*tfinal*.

Typical simulations of basal DSB repair are presented in Figure S3 (upper panels).

**References:**

1. Ma L, Wagner J, Rice JJ, Hu W, Levine AJ, et al. (2005) A plausible model for the digital response of p53 to DNA damage. Proc Natl Acad Sci USA 102: 14266-14271.

2. Loewer A, Batchelor E, Gaglia G, Lahav G (2010) Basal Dynamics of p53 Reveal Transcriptionally Attenuated Pulses in Cycling Cells. Cell 142: 89-100.
